# Somatostatin Inhibits Cell Migration and Reduces Cell Counts of Human Keratinocytes and Delays Epidermal Wound Healing in an *Ex Vivo* Wound Model

**DOI:** 10.1371/journal.pone.0019740

**Published:** 2011-05-11

**Authors:** Matthias Vockel, Simone Pollok, Ute Breitenbach, Ina Ridderbusch, Hans-Jürgen Kreienkamp, Johanna M. Brandner

**Affiliations:** 1 Department of Dermatology and Venerology, University Hospital Hamburg-Eppendorf, Hamburg, Germany; 2 Beiersdorf AG, Hamburg, Germany; 3 Institute for Human Genetics, University Hospital Hamburg-Eppendorf, Hamburg, Germany; 4 Department of Pediatric Surgery, University Hospital Hamburg-Eppendorf, Hamburg, Germany; University of Birmingham, United Kingdom

## Abstract

The peptide hormone somatostatin (SST) and its five G protein-coupled receptors (SSTR1-5) were described to be present in the skin, but their cutaneous function(s) and skin-specific signalling mechanisms are widely unknown. By using receptor specific agonists we show here that the SSTRs expressed in keratinocytes are functionally coupled to the inhibition of adenylate cyclase. In addition, treatment with SSTR4 and SSTR5/1 specific agonists significantly influences the MAP kinase signalling pathway. As epidermal hormone receptors in general are known to regulate re-epithelialization following skin injury, we investigated the effect of SST on cell counts and migration of human keratinocytes. Our results demonstrate a significant inhibition of cell migration and reduction of cell counts by SST. We do not observe an effect on apoptosis and necrosis. Analysis of signalling pathways showed that somatostatin inhibits cell migration independent of its effect on cAMP. Migrating keratinocytes treated with SST show altered cytoskeleton dynamics with delayed lamellipodia formation. Furthermore, the activity of the small GTPase Rac1 is diminished, providing evidence for the control of the actin cytoskeleton by somatostatin receptors in keratinocytes. While activation of all receptors leads to redundant effects on cell migration, only treatment with a SSTR5/1 specific agonist resulted in decreased cell counts. In accordance with reduced cell counts and impaired migration we observe delayed re-epithelialization in an *ex vivo* wound healing model. Consequently, our experiments suggest SST as a negative regulator of epidermal wound healing.

## Introduction

Proliferation and migration of cells play pivotal roles in wound healing as well as in tumorigenesis. During wound closure, the activation and termination of wound healing processes must be tightly regulated to prevent pathological wound responses. Therefore, it is important to identify the signals that direct these cellular processes and elucidate their mechanisms.

Re-epithelialization, which is necessary for wound closure and restoration of barrier function after skin injury, requires directional keratinocyte migration from the wound edges as well as cell proliferation at the wound margins [1], [2]. Both, proliferation and migration of keratinocytes, are controlled by extracellular hormones, providing attractive opportunities for therapeutic intervention [3], [4], [5].

Somatostatin (SST) is a regulatory peptide hormone of 14 amino acids with a wide expression in a variety of tissues [6]. It acts through five different G-protein coupled receptors (SSTR1-5), all of which couple to inhibitory G-proteins of the Gα_i/o_-type. Consequently, many SSTR expressing cells respond to SST treatment by a reduction in cAMP (cyclic adenosine monophosphate) levels [7]. SSTR activation also modulates the MAP (mitogen-activated protein) kinase pathway which is known to have an influence on cell proliferation [8], [9]. In addition, SSTRs hyperpolarize excitable cells through the activation of potassium channels [10] and the inhibition of voltage-gated calcium channels [11]. As has been observed for other G protein-coupled receptors [12], interactions with additional intracellular signalling molecules (e.g. PDZ domain-containing adaptor proteins) modify the subcellular localization and the signalling capabilities of SSTRs [13], [14], [15]. Thus, dependent on the cellular context, SSTRs may not only inhibit the release of neurotransmitters and hormones, but also affect cell proliferation, migration, or the formation of cellular junctions.

We and others have recently provided evidence that SST and its receptors are present in human skin and cultured keratinocytes [14], [16], [17], [18]. SST is mainly found in dendritic cells and Merkel cells [16], [18], [19]. The localization of the five SSTR subtypes was shown in all living layers of the human epidermis by immunohistochemistry with heterogeneous staining intensity and also differences in subcellular localization [17], [18]. Furthermore, in comparison to healthy skin, Hagströmer *et al.* detected an increased immunoreactivity for SSTR4 and SSTR5 in psoriatic epidermis [17]. However, the functional relevance of the various SSTRs and the underlying signalling mechanisms in human keratinocytes are largely unknown except for the involvement of SSTR3 in tight junction composition and function [18].

As endogenous SST agonists (SST14, SST28 and cortistatin) act on all SSTR subtypes with similar efficiency, it has been initially difficult to assign specific functions to receptor subtypes. This has been improved with the advent of specific agonists [20], allowing to dissect the role of individual subtypes more clearly.

Here, we present a systematic functional analysis of the SST/SSTR system in human keratinocytes. Our data confirm on the mRNA level that all five SSTR subtypes are expressed in human skin. In addition, we show for the first time that SST, by inhibiting the activity of Rac1 and influencing lamellipodia formation, is a powerful regulator of keratinocyte migration. Further, we show an inhibitory effect of SST on cell counts independent from apoptosis and necrosis and its influence on the MAP kinase pathway in primary keratinocytes. Our data indicate that these cellular processes might result in an inhibition of wound healing by SST which is consequently shown here for the first time in a porcine *ex-vivo* wound healing model.

## Results

### Expression of all SSTR subtypes in human skin and keratinocytes

**Figure 1:**
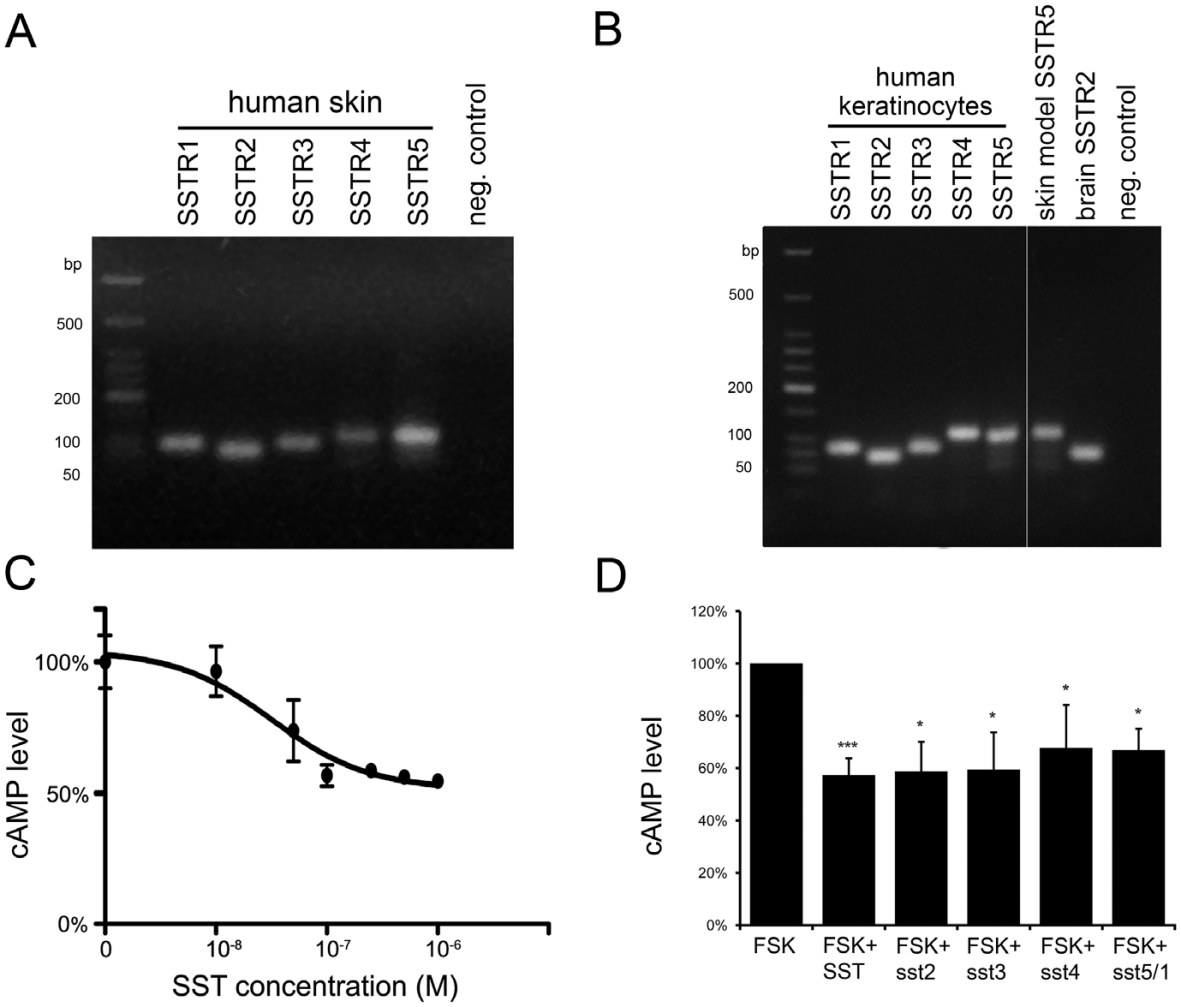
SSTR subtypes are expressed in human skin and are functionally coupled to the cAMP pathway in primary keratinocytes. mRNA isolated from normal human skin (A) and primary human keratinocytes (B) was analyzed for the presence of mRNAs coding for the five somatostatin receptor subtypes SSTR1-5 by RT-PCR using subtype specific primer combinations. In (B) an example for SSTR-expression in epidermal skin equivalents (SSTR5) and in rat brain (SSTR2; positive control) is shown additionally. C: Inhibition of cAMP signalling by increasing concentrations of SST in forskolin-stimulated keratinocytes. Keratinocytes were stimulated with 10 µM forskolin for 10 min without SST (100% cAMP synthesis) or with 10–1000 nM SST. Results are shown as means+/−SD. Data were fitted by non-linear regression using GraphPad Prism software; the calculated IC_50_ value for SST is 32+/−5 nM. D: Effect of different SST receptor agonists on cAMP levels after stimulation of cAMP synthesis by forskolin (FSK, means+/−SEM, * P<0.05, *** P<0.005.). 1 µM SST as well as selective receptor agonists (sst2-sst5/1 at a concentration of 1 µM) inhibited cAMP synthesis, showing that all receptor subtypes couple to cAMP signalling pathways.

For a comprehensive analysis of the SSTR system in human skin, we analyzed samples from human skin by RT-PCR using primers specific for the various SSTR subtypes. mRNAs coding for all five receptor subtypes were detected (Fig. 1A), consistent with the immunohistological observations of a previous report [17]. All five mRNAs were also readily detected in cultured human keratinocytes obtained from neonatal foreskin samples (Fig. 1B). In addition, all receptor mRNAs could be detected in commercially available epidermal skin equivalents (see Fig. 1B for SSTR5 as an example).

### Activation of SSTR subtypes results in adenylate cyclase inhibition

A characteristic feature of SSTRs is to couple to inhibitory G-proteins and therefore to reduce cellular cAMP levels [21]. To clarify whether SSTR subtypes are functionally expressed in human keratinocytes we investigated the effect of SST on cellular cAMP content (after induction of cAMP production by the treatment of cells with forskolin, FSK). Somatostatin elicited a dose dependent decrease in FSK-stimulated cAMP levels, with an IC_50_ of 32 nM (+/−5 nM) (Fig. 1C) which is in agreement with previously published dose response data [10]. Importantly, SST does not affect basal cAMP levels in keratinocytes [18]. Whereas SST is a non-selective agonist for all SSTR subtypes, several subtype specific agonists have been described [20], which were used here to discriminate between the individual subtypes. All specific agonists that were available to us (sst2, sst3, sst4, sst5/1) elicited a decrease in FSK induced cAMP levels, indicating that SSTR2, SSTR3, SSTR4 and either one or both of SSTR1 and SSTR5 - as the corresponding agonist activates both receptors - are coupled to adenylate cyclase inhibition in human keratinocytes, demonstrating their functionality (Fig. 1D).

### SSTR4 and 5/1 activation modulates MAP kinase activity

**Figure 2:**
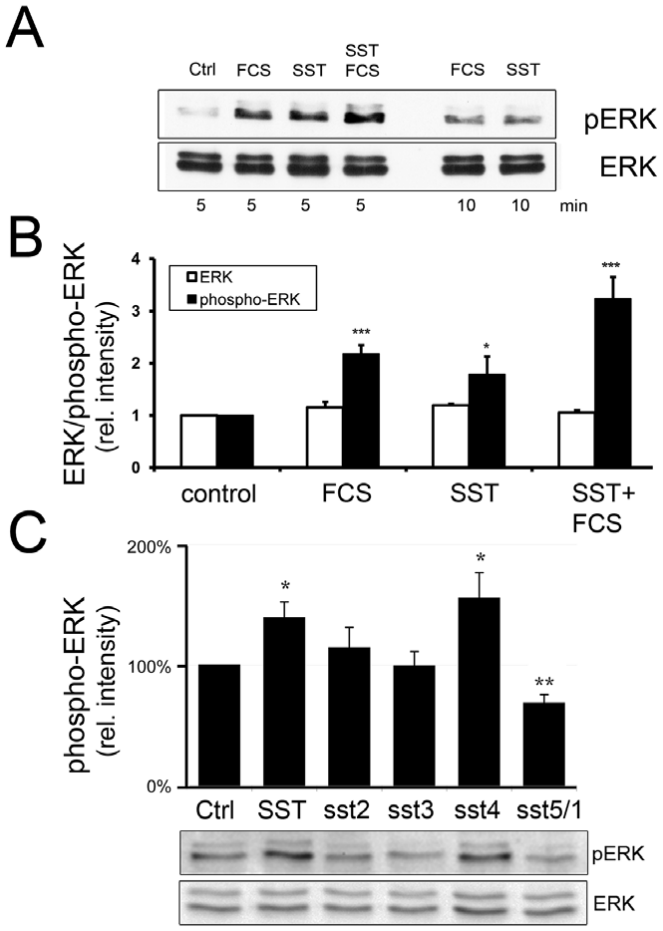
SSTR activation in keratinocytes modulates the MAP kinase pathway. A: MAP kinase activity assay. Cells were treated with 1 µM SST or 5% FCS (or both) for 5 or 10 min and lysates were analyzed with antibodies against ERK1/2 or phospho-ERK1/2 by Western blotting. B: The relative intensities (control value = 1) of 6 experiments after 5 min treatment were quantified and are shown as means +/− SEM (* P<0.05, *** P<0.005.). C: Effect of different SST receptor agonists on ERK phosphorylation (n = 5; * P<0.05, ** P<0.01), a representative blot is shown below. SST as well as the SSTR4-specific agonist significantly induces ERK phosphorylation, while treatment with agonist sst5/1 results in significantly decreased phospho-ERK levels.

To further elucidate somatostatin signaling in keratinocytes, we analyzed the coupling of SSTRs to the activation of MAP kinases. Keratinocytes were treated with SST, fetal calf serum, or a combination of both, and analyzed for the activating ERK (extracellular signal-regulated kinase) phosphorylation using a phospho-ERK1/2 specific antibody. Stimulation with FCS was used as a positive control, as serum growth factors are known to effectively activate ERKs via receptor tyrosine kinases [22], [23]. Here, SST stimulated ERK phosphorylation almost as strongly as FCS, and the combination of FCS and SST led to a further increase in ERK activity (Fig. 2A, B). MAP kinase activation is transient as we observed a prominent phosphorylation 5 min after treatment which was less pronounced after 10 min (Fig. 2A).

By using the subtype specific agonists, the increase in ERK activity can be largely assigned to the action of the SSTR4. Interestingly, the SSTR5/1 specific compound inhibited ERK activation, demonstrating subtype selective activation or inhibition of MAP kinases (Fig. 2C).

Taken together, these data indicate that all SSTR subtypes are expressed in human keratinocytes, and that all of them are functionally coupled to inhibition of adenylate cyclase activity while only SSTR4 (activating) and SSTR5/1 (inactivating) are involved in MAPK signalling. This prompted us to investigate whether SSTRs affect pivotal functions of keratinocytes i.e. proliferation and migration and whether we can find receptor subtype specific differences.

### Cell counts are reduced by activation of SSTR5/1

**Figure 3:**
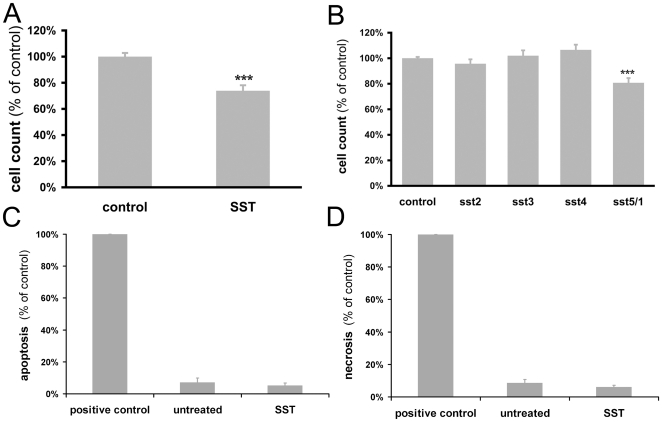
Somatostatin receptor activation reduces keratinocyte cell counts but does not influence apoptosis and necrosis. A: Cells were stimulated with 1 µM SST for 72 h and the effect on cell counts was evaluated. Results are percentages compared to untreated cells (n = 13). B: Effect of selective SSTR agonists on cell counts (n = 4). Only the SSTR5/1-specific agonist inhibits keratinocyte proliferation. C: Effect of 1 µM SST on apoptosis. D: Effect of 1 µM SST on necrosis (positive controls for C and D as included in the assays; positive controls = 100%; n = 3). Results are shown as means+/−SEM, *** P<0.005.

Treatment of actively proliferating keratinocytes with SST resulted in a marked reduction of cell counts 72 hours after application (reduction to 75% compared to non-treated cells, Fig. 3A). Simultaneously, SST treatment had no effect on apoptosis and necrosis (Fig. 3C, D). When we repeated the cell growth experiments using the receptor subtype specific agonists, only the SSTR5/1 specific compound elicited a similar reduction in cell counts. This was consistent with its inhibitory effect on ERK activation (see above). SSTR2, 3 and 4 specific agonists did not show a significant effect (Fig. 3B).

### SST influences keratinocyte migration via all receptor subtypes

**Figure 4:**
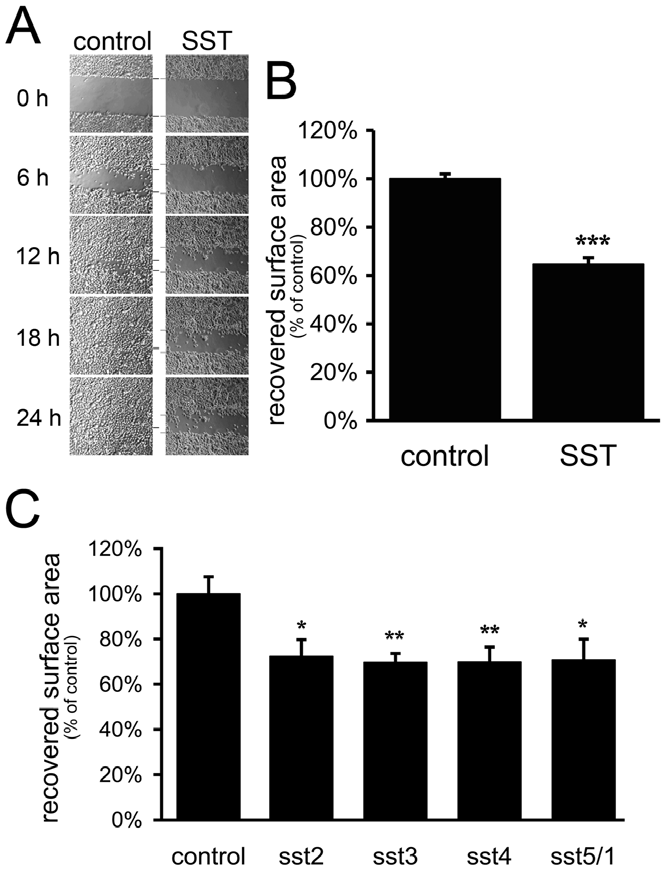
Activation of SST receptors results in the inhibition of keratinocyte migration. A: Keratinocyte monolayers were scratch wounded and the scratched area was examined directly after scratching (0 h) and every 6 hours during an incubation period of 24 h. To prevent proliferative effects, cells were pre-exposed to X-ray irradiation to induce cell cycle arrest. B: Quantification of cell migration in scratch assays after SST stimulation (1 µM SST for 24 h). Data are presented as percentages of the recovered scratch area relative to untreated control cells (n = 7). C: Effect of selective SSTR agonists (1 µM for 24 h) on cell migration compared to untreated cells (n = 5). All subtype-specific agonists tested inhibit keratinocyte migration. Results are shown as means+/−SEM, * P<0.05, ** P<0.01, *** P<0.005.

Cell migration assays were performed with confluent monolayers of human keratinocytes, which were mechanically scratched and subsequently allowed to re-populate the cell-free wounded areas. Cells were pre-exposed to irradiation to exclude any effects of proliferation. While in assays using control keratinocytes the scratched area was closed within 18 h (Fig. 4A), application of SST resulted in a significant delay of scratch area closure, consistent with decreased cell migration (Fig. 4A, B). In contrast to cell counts, which were significantly affected only by the SSTR5/1 specific agonist, migration of keratinocytes was decreased by all four different subtype specific compounds (Fig. 4C). Because we did not observe a significantly higher level of inhibition of migration with somatostatin which activates all five SSTRs compared to the various agonists, a distinct cumulative effect of the agonists is unlikely (Fig. 4B, C).

### Influence of SST on keratinocyte migration is cAMP independent

**Figure 5:**
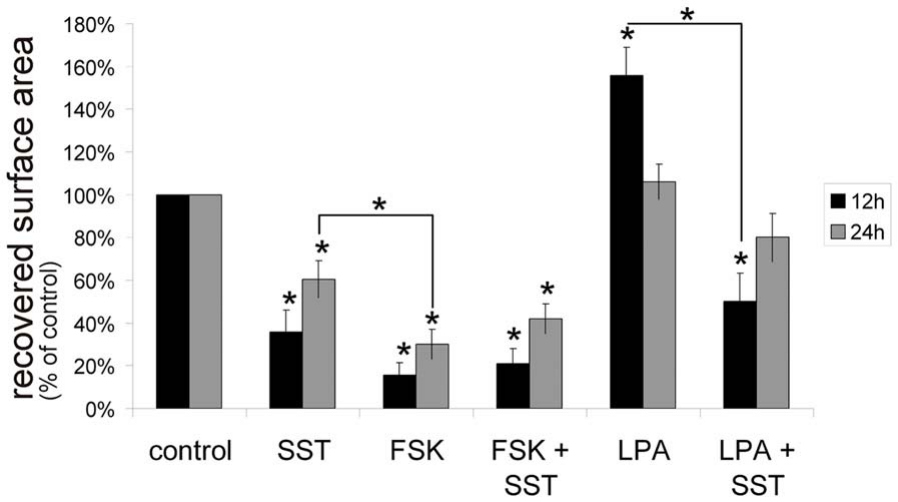
Inhibition of migration by SST is cAMP-independent. Quantification of cell migration in scratch assays after treatment with SST (1 µM), FSK (10 µM) or LPA (5 µM) as well as combinations of these substances. Data are presented as percentages of the recovered scratch area relative to untreated control cells (n = 5) Results are shown as means+/−SEM, * P<0.05, compared to controls; * P<0.05 between different treatment groups.

To elucidate the putative mechanisms for decreased keratinocyte migration after SST application, we first asked whether this effect might be mediated by the cAMP regulation through SST. Therefore, we investigated the influence of forskolin on keratinocyte migration in combination with or without SST. As all five receptor-selective agonists showed redundant anti-migratory effects, we concentrated on the inhibitory effect of the endogenous agonist SST. We observed that the up-regulation of cAMP by forskolin also resulted in a significant inhibition of migration (Fig. 5). This inhibition was even more pronounced than the inhibition obtained by treatment with SST. Combination of SST and forskolin did not enhance the effect of FSK alone. These results strongly suggest that inhibition of migration by SST is not mediated by cAMP.

### SST inhibits lysophosphatidic acid (LPA) induced migration of keratinocytes

Lysophosphatidic acid (LPA), which acts through a different family of G protein-coupled receptors is a stimulator of keratinocyte migration [24]. Therefore we investigated whether somatostatin influences LPA induced migration. Indeed, SST significantly reduces the LPA-induced migration of keratinocytes (Fig. 5), hinting at a common signalling pathway.

### Decreased migration is associated with reduced lamellipodia formation

**Figure 6:**
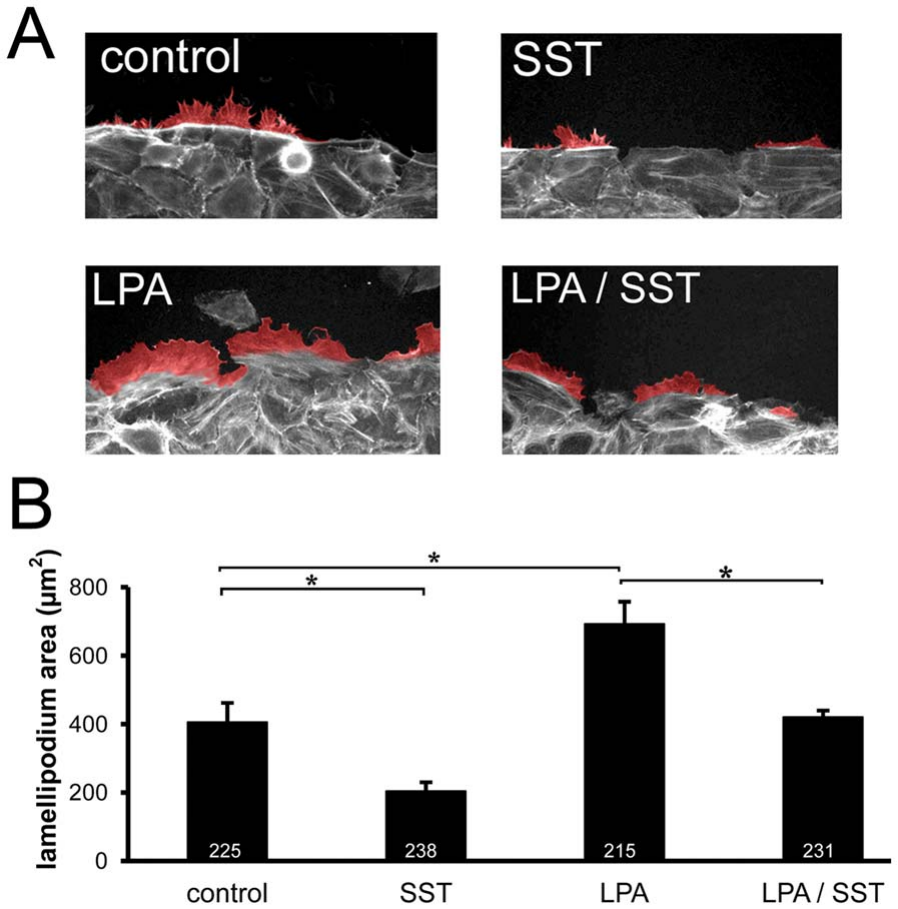
SST delays lamellipodium formation in the early phase of keratinocyte migration. A: Lamellipodium formation in migrating keratinocytes. Cells were scratch wounded, treated as indicated, fixed after 3 h of migration and the actin cytoskeleton was visualized with fluorophor-labelled phalloidin. Lamellipodia are marked by an overlay of red pseudocolor. B: Areas of extending lamellipodia were measured after 3 h for each treatment and compared to control cells (n = 3, total number of analyzed cells is indicated inside bars, means+/−SEM, * P<0.05).

To further investigate which signalling mechanisms in this pathway might mediate this alteration of cell migration, we concentrated on the F-actin based cytoskeleton which is essential for cell motility [25] and which has been shown to be influenced by LPA [26]. In untreated scratched cell monolayers we observed numerous F-actin-rich lamellipodia which extended towards the scratched area. Treatment with SST significantly reduced the area covered by these lamellipodia (Fig. 6). On the other hand, treatment of keratinocytes with LPA results in an increase in lamellipodial area. This increase could be blocked by simultaneous application of SST, clearly demonstrating that SST inhibits lamellipodia formation in migrating keratinocytes.

### SST reduces the amount of active Rac1 during cell migration

**Figure 7:**
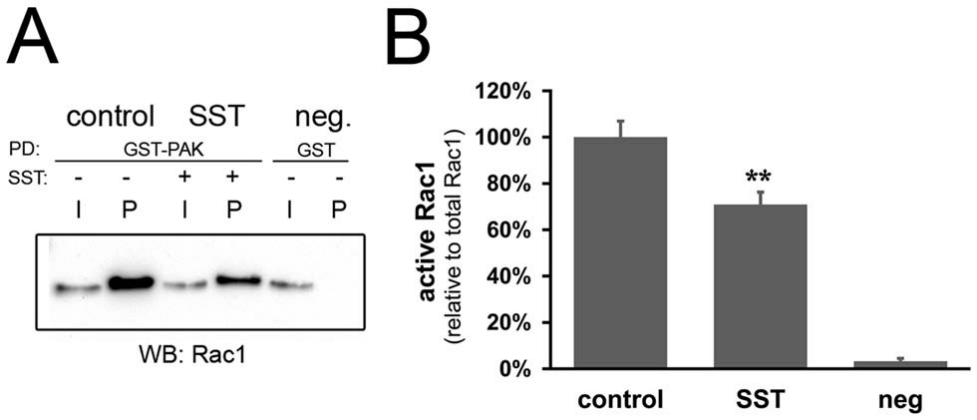
SST decreases Rac1 activity in migrating keratinocytes. A: The amount of active Rac1 was determined by affinity precipitation with purified GST-PAK[PBD]-fusion protein from keratinocyte lysates 3 h after induction of migration. SST treatment reduces active Rac1 compared to untreated cells. Precipitation with GST alone was used as negative control. (I) input, (P) precipitate. B: Relative quantification of Rac1 activity (control = 100%; n = 4). Results are shown as means+/−SEM, ** P<0.01.

Re-arrangements of the actin cytoskeleton have been shown to be mediated by Rho GTPases [27], and formation of lamellipodia in particular by the activity of Rac1 [28], [29]. We therefore investigated whether SST interferes with Rac1 activity during the migration process. Non-proliferating keratinocyte monolayers were again scratched and subsequently treated with or without SST. The active, GTP-bound form of Rac1 was then precipitated from cell lysates using a GST fusion of the Rac binding domain of the typical Rac effector PAK1. We observed a significant reduction in the amount of active Rac1 protein when cells had been treated with SST compared to controls (Fig. 7). Thus, our data demonstrate that activation of SSTRs in keratinocytes leads to an inhibition of Rac1 activity, which is likely to be the cause for the limited lamellipodia formation and cell migration.

### SST delays epidermal re-epithelialization in an *ex vivo* wound healing model

**Figure 8:**
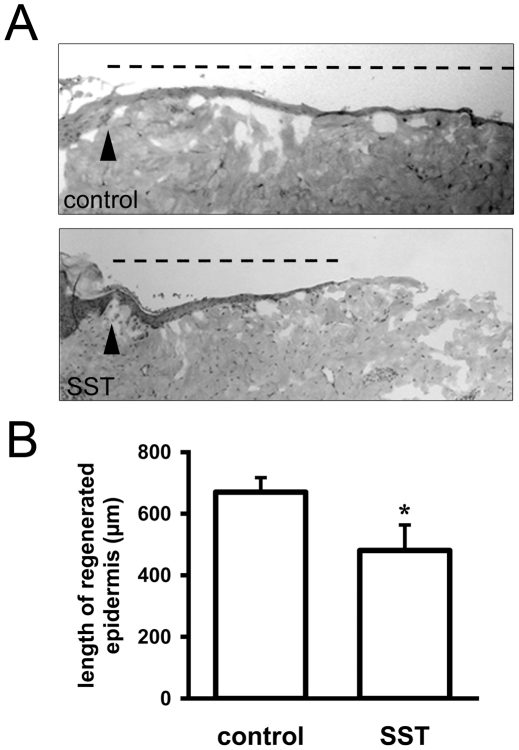
SST delays epidermal wound healing in a porcine *ex vivo* model. A: *Ex vivo* wound healing models from porcine ear skin were treated with SST for 48 h and compared to control models. Examples for hematoxylin/eosin stainings of control (upper picture) and SST treated (lower picture) models. The wound margin is indicated by an arrowhead and the regenerated epidermis is depicted by a dashed line above the model. While the control model shows complete re-epithelialization, application of SST inhibits wound closure. B Quantification of epidermal wound healing. Re-epithelialization was measured at both wound margins by an investigator blind to the experimental conditions. Data are depicted as mean +/− SEM; *, p<0.05. n = 6.

As both keratinocyte proliferation and migration were inhibited by SSTR activation, we next determined effects of SST on epidermal re-epithelialization in a porcine *ex vivo* wound healing model. We compared wound closure in untreated and SST-treated wound models 48 h post-wounding and observed that epidermal wound closure is delayed in SST-treated models (Fig. 8A). Quantitative evaluation of the healing rates shows that re-epithelialization is significantly reduced in the presence of SST (Fig. 8B).

## Discussion

In agreement with previous studies from our and other laboratories, our data firmly establish the presence of the somatostatin receptor system in human skin and in cultured human keratinocytes. Besides supporting on a molecular level the findings of Hagströmer *et al.* [17], who have shown the presence of all five SSTR subtypes by immunohistochemistry, we now present for the first time evidence that all of them are functionally coupled to effector proteins such as the adenylate cyclase and – for some subtypes - MAP kinases.

It was shown before that the ligand somatostatin itself is expressed in the epidermis primarily in Merkel cells as well as in dendritic cells [16], [18], [19]. In addition, it was demonstrated that nerve fibres in the skin are positive for somatostatin [30]. Senapati et al. [31] determined the concentration of somatostatin in rat skin to be 1–3 nM, i.e. in the range of Kd-values reported for SSTR1-5. Local concentrations at secretion sites may be even higher.

These findings prompted us to ask to what extent SST contributes to the regulation of important epidermal functions, i.e. the cell growth and migration of keratinocytes which are essential elements of the wound healing process but also for tumorigenesis.

Cell counts are specifically reduced by the application of SST and the SSTR5/1 specific agonist. Because we did not see an influence of SST on apoptosis and necrosis we assume that reduced cell counts origin in a reduced cell proliferation. However, we can not exclude a further effect on cellular senescence. Interestingly, SSTR5/1 is the very agonist which was able to reduce the amount of the activated form of ERK. A negative influence on cell proliferation is a common characteristic of SST, which has been demonstrated e.g. in different carcinoma cell lines [32], [33], but has never been shown for keratinocytes. As the agonist used activates both SSTR1 and 5 we were not able to distinguish between these two receptors, but the effect is likely to be mediated by SSTR5, as this subtype has been described to inhibit proliferation via MAP kinases [34]. The fact that the SSTR2 specific agonist does not elicit inhibition of cell growth may seem surprising, giving the large body of literature linking this receptor to antiproliferative signalling (e.g. [33]). However, it should be noted that such effects were observed mostly in tumor cells which overexpress the SSTR2. In keratinocytes, the SSTR2 is likely to be expressed at physiological levels, which may not be sufficient to inhibit proliferation. Of note, Haegerstrand et al. [35] did not observe an influence of 0.1 µM somatostatin on cell count of primary keratinocytes. However, the cell culture conditions used in their experiments were completely different to ours and included mouse feeder cells, BSA and high concentrations of EGF which might influence the results. In addition, the discrepancy could originate in different concentrations of somatostatin as we used 1 µM.

In addition to the effects on cell growth we could show for the first time an inhibition of cell migration by SST in human keratinocytes. Of note, in contrast to the restricted influence of SST on cell counts only via SSTR5/1, migration is inhibited by the activation of all receptor subtypes, indicating a redundancy of the receptors for this function. Our data suggest that inhibition of cell migration by somatostatin is a common cellular feature, as it was also found in cells of neuronal origin [36], [37]: Consistent with our observations, Pola and colleagues observed that all subtype selective SSTR agonists significantly reduced cell migration in neuroblastoma cells [37]. The redundancy of SSTR subtypes for migration in different cellular systems might reflect their importance for this cellular process. We assume that the specific complement of receptor subtypes expressed in keratinocytes enables the cell to shape a specific response e.g. in the wound healing process. Thus either SSTR1 or SSTR5 will be necessary to inhibit proliferation, while the other subtypes may also contribute to the regulation of migration.

Even though all receptor subtypes lead to a decrease of forskolin-stimulated cAMP levels and to a decrease in migration, our experiments using forskolin to increase cellular cAMP levels show that the influence of SST on migration is independent from its effect on cAMP. As already described by McCawley et al [38], elevation of cAMP levels by forskolin efficiently reduces cell migration. This reduction was not affected by simultanous treatment with SST. Our data fit well with findings of Chen *et al.*, (2002) who described that activation of the β_2_-adrenergic receptor inhibits keratinocyte migration via a cAMP-independent mechanism [39]. The β_2_-adrenergic receptor is positively coupled to cAMP generation via the stimulatory G-protein Gα_s_ [4] and Chen *et al.* proposed that the decrease of migration is mediated by inhibition of MAP kinase signalling. However, even though we have shown here that the MAPK pathway is influenced by SST, it is unlikely that this is the cause for the decrease of migration observed in our studies. Migration is inhibited by all 5 receptors of SST while ERK activation is only inhibited by SSTR1/5 and even increased by SSTR4.

At present we think that SSTRs affect cell migration and lamellipodia formation through modulation of Rho GTPase signalling. This could either be achieved through an inhibition of Rho-family-specific guanine nucleotide exchange factors (RhoGEFs) or an activation of GTPase activating proteins (RhoGAPs). Members of both protein families have been described to be activated by subunits of the heterotrimeric G-proteins (e.g. [40], [41]) and in some cases interact directly with receptors via C-terminal PDZ domain binding motifs [42]. Interestingly, such motifs are also present in SSTRs [14]. We observed a decreased activation of Rac1 by SST; this Rho GTPase has been shown to be involved in directed migration and reorganization of the cellular actin cytoskeleton [27]. Interestingly, Tscharntke and colleagues [43] showed that Rac1 deletion in primary keratinocytes or transgenic mice leads to reduced cell migration and re-epithelialization. Furthermore, deletion of Rac1 in fibroblasts inhibits wound healing *in vivo* [44]. The induction of lamellipodia formation by the phospholipid LPA is also dependent on Rac1 activation [29]. In the presence of SST, we observed a reduction in LPA-induced lamellipodia formation in human keratinocytes. Therefore, we suggest that SST influences Rac1 activation and reorganization of the cytoskeleton. However, one has to keep in mind that LPA can bind to several receptors and can influence besides Rac1 also cAMP levels, ERK, phospholipase C and intracellular Ca^2+^. Furthermore it can also result in gap junction closure and tight junction (TJ) opening [45], [46]. Consequently, we can not exclude that SST might also be involved in additional signalling pathways. Of note, we have previously shown that SST can increase TJ barrier function via the human SSTR3 [18], [47].

As keratinocyte proliferation and migration are essential processes in wound healing, we investigated the influence of SST on re-epithelialization in an *ex vivo* wound healing model. In accordance with the decrease in proliferation and migration in cultured keratinocytes we observed a delay of re-epithelialization in our wound healing model. Interestingly, Waddell *et al.*, described a negative influence of the therapeutic SSTR agonist octreotide (Sandostatin®) on wound breaking strength in rat skin [48]. The authors attribute this effect to an inhibition of trophic hormones following a 7-day subcutanous injection of octreotide. In contrast, we could show a local action of SST in epidermal wound healing and propose a direct effect of SST on epidermal keratinocytes.

While the effects of activating signals (e.g. growth factors) on wound healing are well studied, the influence of negative regulators is largely unknown [2], [49]. However, after induction by positive regulators it is important to control the fundamental changes in human keratinocyte activity by limiting factors (such as somatostatin). These regulators are required to self-limit the wound repair process to ensure an orchestrated closure of the wound. Of note, there is growing evidence that wounding promotes epidermal tumorigenesis [50], [51] and several authors have hypothesized that “cancer is an overhealing wound” (for review see [52]) which also points to a role of SST agonists in the therapy of skin cancer. An interesting question is how somatostatin and SSTR expression changes during wound regeneration. A decrease in epidermal concentrations of SST has been reported during early phases of wound healing in rat skin [31]. The authors do not discuss the relevance of this depletion, but it is conceivable that this decrease allows efficient keratinocyte migration and proliferation during wound repair. To this end, it would be interesting to test whether somatostatin levels do increase in later stages of the wound healing process. Therefore, in future experiments it will be of great interest to investigate how SSTR and SST expression is regulated in normal human wound healing and chronic wounds and also to specifically elucidate the potential of SSTR antagonists in these wounds.

In conclusion, our data show for the first time receptor subtype-specific signal transduction pathways of SST in human keratinocytes and its influence on migration and cell counts and, consequently, re-epithelialization of cutaneous wounds.

## Materials and Methods

### Antibodies, cDNAs, primers and reagents

Rabbit anti-ERK (#9102) and mouse anti-phospho-ERK (#9106) were purchased from Cell Signaling (Danvers, USA), fluorophor-labelled phalloidin (#MFP-A2283) was from Mobitec (Göttingen, Germany) and mouse anti-Rac1 (#610650) from BD Biosciences (San Jose, USA). Somatostatin was purchased from Bachem (Weil am Rhein, Germany, #H-1490), Forskolin (FSK, #F6886) and lysophosphatidic acid (LPA, #L7260) were from Sigma. Whole skin and brain cDNA was purchased from Invitrogen (Karlsruhe, Germany). Subtype specific SSTR agonists (sst2: L-779,976, sst3: L-796,778, sst4: L-803,087 and sst5/1: L-817,818) were obtained from Merck Research Laboratories (Rahway, New Jersey). The binding affinities of somatostatin and the SSTR agonists are: Somatostatin: 0.5–1.6 nM for SSTR1-5; sst2: 0.05 nM for SSTR2; sst3: 24 nM for SSTR3; sst4: 0.7 nM for SSTR4; sst5/1: 0.4 nM for SSTR5, 3.3 nM for SSTR1 [20], [53]. Values were determined in receptor-overexpressing cell lines and due to lower efficacies in primary cells, concentrations in micro-molar range were chosen for experiments. The receptor-specific agonists exhibit 100-fold to 10,000-fold selectivity against the other SSTR subtypes. SSTR subtype specific primers (SSTR1: Hs00265617_s1, SSTR2: Hs00265624_s1, SSTR3: Hs00265633_s1, SSTR4: Hs01566620_s1, SSTR5: Hs00265647_s1) were purchased from Applied Biosystems (Darmstadt, Germany).

### Cell culture

Normal human skin used for the cultivation of primary keratinocytes was obtained during the routine clinical removal of neonatal foreskin. Their usage was approved by the local medical ethics committee (060900). Isolated cells were cultured in keratinocyte growth medium (KGM; Medium 154, Cascade Biologics, Karlsruhe, Germany) supplemented with 0.07 mM Ca^2+^ using a modified protocol of Rheinwald and Green [54].

### Determination of cell counts

For the determination of cell counts, keratinocytes were seeded in 6 well plates (density: 50,000 cells/well) and treated with 1 µM SST or selective SSTR agonists for 72 h. Cells were trypsinized and cell numbers were determined by using a hemocytometer.

### Determination of apoptosis and necrosis

For determination of cell death, keratinocytes were seeded into 24-well plates at a density of 650,000 cells/well and treated with 1 µM SST for 72 hours. Afterwards, apoptosis was measured by applying the Cell Death Detection ELISA kit (Roche, Mannheim, Germany) and necrosis by applying the Cytotoxicity Detection ELISA kit (Roche, Mannheim, Germany).

### Cell scratch assay

For *in vitro* migration assays, keratinocytes were seeded in Collagen I-coated wells at a density of 100,000 cells/well. After reaching confluency, cells were irradiated for 20 min with 200 keV (0.8 Gy/min) to induce cell cycle arrest. The cell monolayer was wounded using a sterile pipette tip and washed twice with PBS to remove cell debris. Then, KGM with 1 µM SST, 1 µM selective SSTR agonists, 10 µM FSK or 5 µM LPA was added. The wound area was photographed with phase contrast at marked positions (3 different fields per well in triplicate). Cells were allowed to migrate for 12 and 24 h at 37°C and the same fields were photographed again. Scratched areas were measured with ImageJ software (NIH, Bethesda, USA) and recovered surface area over 12 and 24 h was calculated compared to untreated cells.

### Measurement of lamellipodium areas

Measurement of lamellipodium areas was done by immunofluorescent visualization of actin-rich cell protrusions. Keratinocytes were grown on coverslips to confluency and scratch wounded as described above. Cells were treated with either 1 µM SST, 5 µM LPA or both. After 3 h of migration, cells were fixed (4% formaldehyde in PBS) and permeabilized (0.1% Triton X-100 in PBS). After blocking (3% BSA), cells were incubated with fluorophor-labelled phalloidin and counterstained with DAPI. After mounting, the actin cytoskeleton was visualized with an Axiovert 135 epifluorescence microscope (Zeiss, Göttingen, Germany) and the area of lamellipodia extending into the wound surface was measured with ImageJ software.

### RNA isolation and RT-PCR

Total RNA from keratinocytes was isolated using Tri reagent (Sigma) and a subsequent purification using RNeasy columns (Qiagen, Hilden, Germany). cDNA was generated with iScript cDNA synthesis kit (Bio-Rad, Munich, Germany) and PCR was performed as described [55]. Preparations of RNA template without reverse transcriptase were used as negative controls.

### Cyclic AMP assay

Cells were seeded in 96-well dishes at a density of 25,000 cells/well. Before stimulation, cells were preincubated for 30 min with the phosphodiesterase inhibitor isobutylmethylxanthine (500 µM in KGM) to prevent cAMP degradation. The cells were incubated for 10 min with 10 µM forskolin in the absence or presence of different concentrations of somatostatin. Intracellular cyclic AMP levels were determined using the HitHunter cAMP XS+ kit according to the manufacturer's instructions (GE Healthcare, Munich, Germany).

### Measurement of MAP kinase activity

Primary keratinocytes were stimulated with 1 µM SST, 5% FCS or both and incubated for 5 and 10 minutes at 37°C. After washing with PBS, cells were lyzed with lysis buffer (50 mM Tris/HCl; pH 8.0, 150 mM NaCl, 1% (v/v) NP-40, 0.5% (w/v) Na-deoxycholate, 5 mM EDTA, 0.1% SDS). Lysates were cleared by centrifugation and subjected to SDS-PAGE. Amounts of total and active ERK (extracellular signal-regulated kinase)/MAP kinase were determined with ERK1/2 and phospho-ERK1/2 antibodies by Western blotting. Quantifications of signal intensities were done with a ChemiDoc XRS imager and Quantity One software (Bio-Rad).

### Active Rac1 precipitation assay

Active (GTP-bound) Rac1 was precipitated from keratinocyte lysates with an immobilized glutathione-S-transferase (GST) fusion protein of the purified Rac1 binding domain of the p21 activated kinase PAK1(PAK[PBD]; [56]). The GST-PAK fusion protein was expressed and isolated from *E. coli* and bound to glutathione sepharose (GE Healthcare); GST alone was used as a control. Confluent primary keratinocytes were extensively scratched so that a large percentage of cells was localized at wound edges, and lyzed after 3 hours of migration with lysis buffer. Lysates were cleared by centrifugation and GST-PAK-beads were added to cleared lysates and incubated for 1 h at 4°C on a rotator. The beads were washed three times with lysis buffer and bound proteins were separated by 12% SDS-PAGE. GST-PAK bound (active) and total cellular Rac1 were detected by Western blotting using a monoclonal antibody specific for Rac1 (BD Biosciences). Signal intensities were quantified with a ChemiDoc XRS imager and Quantity One software (Bio-Rad).

### *Ex vivo* wound healing models

Punch biopsies with a diameter of 6 mm were taken from porcine ear skin. Subsequently another 3 mm punch biopsy including epidermis and the upper dermis was excised, resulting in a central wound. The biopsies were placed dermis down on gauze in culture dishes filled with Dulbecco's modified Eagle's medium supplemented with 2% fetal calf serum, hydrocortisone, penicillin and streptomycin. The resulting models were incubated at air-liquid interface at 37°C with the application of 5 µl PBS or SST (2 µM) into the central wound directly and 24 h after wounding. After 48 hours, the samples were snap-frozen in isopentane precooled with liquid nitrogen and stored at −80°C. Re-epithelialization was evaluated in hematoxylin and eosin-stained cryostat sections by measuring the length of regenerated epidermis with an axiophot II microscope and openlab software 2.0.9 (Improvision, Coventry, UK) light microscopy [57], [58].

### Statistical analysis

Data are presented as mean values ± standard deviation or ± standard error of mean (see figure legends). Data sets were checked for normal distribution (Shapiro-Wilk test) and equal variance. When possible, p-values were determined by Student's t-test. Otherwise (MAPK-Phosphorylation, Proliferation with Agonists, Rac-Pulldown), Mann-Whitney U test was performed. Differences were considered statistically significant with a p-value less than 0.05.

## References

[1] Gurtner GC, Werner S, Barrandon Y, Longaker MT (2008). Wound repair and regeneration.. Nature.

[2] Martin P (1997). Wound healing-aiming for perfect skin
regeneration.. Science.

[3] Chernyavsky AI, Arredondo J, Wess J, Karlsson E, Grando SA (2004). Novel signaling pathways mediating reciprocal control of
keratinocyte migration and wound epithelialization through M3 and M4
muscarinic receptors.. J Cell Biol.

[4] Pullar CE, Grahn JC, Liu W, Isseroff RR (2006). Beta2-adrenergic receptor activation delays wound
healing.. Faseb J.

[5] Taboubi S, Milanini J, Delamarre E, Parat F, Garrouste F (2007). G alpha(q/11)-coupled P2Y2 nucleotide receptor inhibits human
keratinocyte spreading and migration.. Faseb J.

[6] Patel YC (1999). Somatostatin and its receptor family.. Front Neuroendocrinol.

[7] Patel YC, Greenwood MT, Warszynska A, Panetta R, Srikant CB (1994). All five cloned human somatostatin receptors (hSSTR1-5) are
functionally coupled to adenylyl cyclase.. Biochem Biophys Res Commun.

[8] Cattaneo MG, Amoroso D, Gussoni G, Sanguini AM, Vicentini LM (1996). A somatostatin analogue inhibits MAP kinase activation and cell
proliferation in human neuroblastoma and in human small cell lung carcinoma
cell lines.. FEBS Lett.

[9] Csaba Z, Dournaud P (2001). Cellular biology of somatostatin receptors.. Neuropeptides.

[10] Kreienkamp HJ, Hönck HH, Richter D (1997). Coupling of rat somatostatin receptor subtypes to a G-protein
gated inwardly rectifying potassium channel (GIRK1).. FEBS Lett.

[11] Meriney SD, Gray DB, Pilar GR (1994). Somatostatin-induced inhibition of neuronal Ca2+ current
modulated by cGMP-dependent protein kinase.. Nature.

[12] Hall RA, Premont RT, Chow CW, Blitzer JT, Pitcher JA (1998). The beta2-adrenergic receptor interacts with the
Na+/H+-exchanger regulatory factor to control Na+/H+
exchange.. Nature.

[13] Christenn M, Kindler S, Schulz S, Buck F, Richter D (2007). Interaction of brain somatostatin receptors with the PDZ domains
of PSD-95.. FEBS Lett.

[14] Liew CW, Vockel M, Glassmeier G, Brandner JM, Fernandez-Ballester GJ (2009). Interaction of the human somatostatin receptor 3 with the
multiple PDZ domain protein MUPP1 enables somatostatin to control
permeability of epithelial tight junctions.. FEBS Lett.

[15] Wente W, Efanov AM, Treinies I, Zitzer H, Gromada J (2005). The PDZ/coiled-coil domain containing protein PIST modulates
insulin secretion in MIN6 insulinoma cells by interacting with somatostatin
receptor subtype 5.. FEBS Lett.

[16] Gaudillere A, Misery L, Bernard C, Souchier C, Claudy A (1997). Presence of somatostatin in normal human
epidermis.. Br J Dermatol.

[17] Hagströmer L, Emtestam L, Stridsberg M, Talme T (2006). Expression pattern of somatostatin receptor subtypes 1–5 in
human skin: an immunohistochemical study of healthy subjects and patients
with psoriasis or atopic dermatitis.. Exp Dermatol.

[18] Vockel M, Breitenbach U, Kreienkamp HJ, Brandner JM (2010). Somatostatin regulates tight junction function and composition in
human keratinocytes.. Exp Dermatol.

[19] Fantini F, Johansson O (1995). Neurochemical markers in human cutaneous Merkel cells. An
immunohistochemical investigation.. Exp Dermatol.

[20] Rohrer SP, Birzin ET, Mosley RT, Berk SC, Hutchins SM (1998). Rapid identification of subtype-selective agonists of the
somatostatin receptor through combinatorial chemistry.. Science.

[21] Weckbecker G, Lewis I, Albert R, Schmid HA, Hoyer D (2003). Opportunities in somatostatin research: biological, chemical and
therapeutic aspects.. Nat Rev Drug Discov.

[22] Chang L, Karin M (2001). Mammalian MAP kinase signalling cascades.. Nature.

[23] Katz M, Amit I, Yarden Y (2007). Regulation of MAPKs by growth factors and receptor tyrosine
kinases.. Biochim Biophys Acta.

[24] Sauer B, Vogler R, Zimmermann K, Fujii M, Anzano MB (2004). Lysophosphatidic acid interacts with transforming growth
factor-beta signaling to mediate keratinocyte growth arrest and
chemotaxis.. J Invest Dermatol.

[25] Pollard TD, Borisy GG (2003). Cellular motility driven by assembly and disassembly of actin
filaments.. Cell.

[26] Khurana S, Tomar A, George S, Wang Y, Siddiqui M (2008). Autotaxin and lysophosphatidic acid stimulate intestinal cell
motility by redistribution of the actin modifying protein villin to the
developing lamellipodia.. Exp Cell Res.

[27] Etienne-Manneville S, Hall A (2002). Rho GTPases in cell biology.. Nature.

[28] Ridley AJ, Paterson HF, Johnston CL, Diekmann D, Hall A (1992). The small GTP-binding protein rac regulates growth factor-induced
membrane ruffling.. Cell.

[29] Van Leeuwen FN, Olivo C, Grivell S, Giepmans BN, Collard JG (2003). Rac activation by lysophosphatidic acid LPA1 receptors through
the guanine nucleotide exchange factor Tiam1.. J Biol Chem.

[30] Johansson O, Vaalasti A (1987). Immunohistochemical evidence for the presence of
somatostatin-containing sensory nerve fibres in the human
skin.. Neurosci Lett.

[31] Senapati A, Anand P, McGregor GP, Ghatei MA, Thompson RP (1986). Depletion of neuropeptides during wound healing in rat
skin.. Neurosci Lett.

[32] Benali N, Cordelier P, Calise D, Pages P, Rochaix P (2000). Inhibition of growth and metastatic progression of pancreatic
carcinoma in hamster after somatostatin receptor subtype 2 (sst2) gene
expression and administration of cytotoxic somatostatin analog
AN-238.. Proc Natl Acad Sci U S A.

[33] Bousquet C, Guillermet J, Vernejoul F, Lahlou H, Buscail L (2004). Somatostatin receptors and regulation of cell
proliferation.. Dig Liver Dis.

[34] Cordelier P, Esteve JP, Bousquet C, Delesque N, O'Carroll AM (1997). Characterization of the antiproliferative signal mediated by the
somatostatin receptor subtype sst5.. Proc Natl Acad Sci U S A.

[35] Haegerstrand A, Jonzon B, Dalsgaard CJ, Nilsson J (1989). Vasoactive intestinal polypeptide stimulates cell proliferation
and adenylate cyclase activity of cultured human
keratinocytes.. Proc Natl Acad Sci U S A.

[36] Cattaneo MG, Gentilini D, Vicentini LM (2006). Deregulated human glioma cell motility: inhibitory effect of
somatostatin.. Mol Cell Endocrinol.

[37] Pola S, Cattaneo MG, Vicentini LM (2003). Anti-migratory and anti-invasive effect of somatostatin in human
neuroblastoma cells: involvement of Rac and MAP kinase
activity.. J Biol Chem.

[38] McCawley LJ, Li S, Benavidez M, Halbleib J, Wattenberg EV (2000). Elevation of intracellular cAMP inhibits growth factor-mediated
matrix metalloproteinase-9 induction and keratinocyte
migration.. Mol Pharmacol.

[39] Chen J, Hoffman BB, Isseroff RR (2002). Beta-adrenergic receptor activation inhibits keratinocyte
migration via a cyclic adenosine monophosphate-independent
mechanism.. J Invest Dermatol.

[40] Bartolome R, Wright N, Molina-Ortiz I, Sanchez-Luque F, Teixido J (2008). Activated G(alpha)13 impairs cell invasiveness through
p190RhoGAP-mediated inhibition of RhoA activity.. Cancer Res.

[41] Lutz S, Freichel-Blomquist A, Yang Y, Rumenapp U, Jakobs KH (2005). The guanine nucleotide exchange factor p63RhoGEF, a specific link
between Gq/11-coupled receptor signaling and RhoA.. J Biol Chem.

[42] Yamada T, Ohoka Y, Kogo M, Inagaki S (2005). Physical and functional interactions of the lysophosphatidic acid
receptors with PDZ domain-containing Rho guanine nucleotide exchange factors
(RhoGEFs).. J Biol Chem:.

[43] Tscharntke M, Pofahl R, Chrostek-Grashoff A, Smyth N, Niessen C (2007). Impaired epidermal wound healing in vivo upon inhibition or
deletion of Rac1.. J Cell Sci.

[44] Liu S, Kapoor M, Leask A (2009). Rac1 expression by fibroblasts is required for tissue repair in
vivo.. Am J Pathol.

[45] Mills GB, Moolenaar WH (2003). The emerging role of lysophosphatidic acid in
cancer.. Nat Rev Cancer.

[46] Meyer zu Heringdorf D, Jakobs KH (2007). Lysophospholipid receptors: signalling, pharmacology and
regulation by lysophospholipid metabolism.. Biochim Biophys Acta.

[47] Vockel M, Breitenbach U, Kreienkamp HJ, Brandner JM (2010). Somatostatin regulates Tight Junction function and composition in
human keratinocytes.. Exp Dermato.

[48] Waddell BE, Calton WC, Steinberg SR, Martindale RG (1997). The adverse effects of octreotide on wound healing in
rats.. Am Surg.

[49] Barrientos S, Stojadinovic O, Golinko MS, Brem H, Tomic-Canic M (2008). Growth factors and cytokines in wound healing.. Wound Repair Regen.

[50] Kasper M, Jaks V, Are A, Bergstrom A, Schwager A (2011). Wounding enhances epidermal tumorigenesis by recruiting hair
follicle keratinocytes.. Proc Natl Acad Sci U S A.

[51] Wong SY, Reiter JF (2011). From the Cover: Wounding mobilizes hair follicle stem cells to
form tumors.. Proc Natl Acad Sci U S A.

[52] Schafer M, Werner S (2008). Cancer as an overhealing wound: an old hypothesis
revisited.. Nat Rev Mol Cell Biol.

[53] Patel YC, Srikant CB (1994). Subtype selectivity of peptide analogs for all five cloned human
somatostatin receptors (hsstr 1–5).. Endocrinology.

[54] Rheinwald JG, Green H (1975). Serial cultivation of strains of human epidermal keratinocytes:
the formation of keratinizing colonies from single cells.. Cell.

[55] Brandner JM, Kief S, Grund C, Rendl M, Houdek P (2002). Organization and formation of the tight junction system in human
epidermis and cultured keratinocytes.. Eur J Cell Biol.

[56] Benard V, Bohl BP, Bokoch GM (1999). Characterization of rac and cdc42 activation in
chemoattractant-stimulated human neutrophils using a novel assay for active
GTPases.. J Biol Chem.

[57] Brandner JM, Houdek P, Quitschau T, Siemann-Harms U, Ohnemus U (2006). An ex-vivo model to evaluate dressings & drugs for wound
healing. Example: Influence of lucilia sericata extracts on wound healing
progress.. EWMA J.

[58] Pollok S, Pfeiffer AC, Lobmann R, Wright CS, Moll I (2010). Connexin 43 mimetic peptide Gap27 reveals potential differences
in the role of Cx43 in wound repair between diabetic and non-diabetic
cells.. J Cell Mol Med.

